# An MRI-based grading system for preoperative risk estimation of positive surgical margin after radical prostatectomy

**DOI:** 10.1186/s13244-023-01516-4

**Published:** 2023-10-23

**Authors:** Lili Xu, Gumuyang Zhang, Daming Zhang, Jiahui Zhang, Xiaoxiao Zhang, Xin Bai, Li Chen, Qianyu Peng, Yu Xiao, Hao Wang, Zhengyu Jin, Hao Sun

**Affiliations:** 1 Department of Radiology, State Key Laboratory of Complex Severe and Rare Disease, Peking Union Medical College Hospital, Peking Union Medical College, Chinese Academy of Medical Sciences, No.1 Shuaifuyuan, Wangfujing Street, Dongcheng District, Beijing, 100730 China; 2 National Center for Quality Control of Radiology, No.1 Shuaifuyuan, Wangfujing Street, Dongcheng District, Beijing, 100730 China; 3 Department of Pathology, Peking Union Medical College Hospital, Peking Union Medical College, Chinese Academy of Medical Sciences, No.1 Shuaifuyuan, Wangfujing Street, Dongcheng District, Beijing, 100730 China

**Keywords:** Prostatic neoplasms, Magnetic resonance imaging, Margins of excision, Risk assessment

## Abstract

**Objective:**

To construct a simplified grading system based on MRI features to predict positive surgical margin (PSM) after radical prostatectomy (RP).

**Methods:**

Patients who had undergone prostate MRI followed by RP between January 2017 and January 2021 were retrospectively enrolled as the derivation group, and those between February 2021 and November 2022 were enrolled as the validation group. One radiologist evaluated tumor-related MRI features, including the capsule contact length (CCL) of lesions, frank extraprostatic extension (EPE), apex abutting, etc. Binary logistic regression and decision tree analysis were used to select risk features for PSM. The area under the curve (AUC), sensitivity, and specificity of different systems were calculated. The interreader agreement of the scoring systems was evaluated using the kappa statistic.

**Results:**

There were 29.8% (42/141) and 36.4% (32/88) of patients who had PSM in the derivation and validation cohorts, respectively. The first grading system was proposed (mrPSM1) using two imaging features, namely, CCL ≥ 20 mm and apex abutting, and then updated by adding frank EPE (mrPSM2). In the derivation group, the AUC was 0.705 for mrPSM1 and 0.713 for mrPSM2. In the validation group, our grading systems showed comparable AUC with Park et al.’s model (0.672–0.686 vs. 0.646, *p* > 0.05) and significantly higher specificity (0.732–0.750 vs. 0.411, *p* < 0.001). The kappa value was 0.764 for mrPSM1 and 0.776 for mrPSM2. Decision curve analysis showed a higher net benefit for mrPSM2.

**Conclusion:**

The proposed grading systems based on MRI could benefit the risk stratification of PSM and are easily interpretable.

**Critical relevance statement:**

The proposed mrPSM grading systems for preoperative prediction of surgical margin status after radical prostatectomy are simplified compared to a previous model and show high specificity for identifying the risk of positive surgical margin, which might benefit the management of prostate cancer.

**Key points:**

• CCL ≥ 20 mm, apex abutting, and EPE were important MRI features for PSM.

• Our proposed MRI-based grading systems showed the possibility to predict PSM with high specificity.

• The MRI-based grading systems might facilitate a structured risk evaluation of PSM.

**Graphical Abstract:**

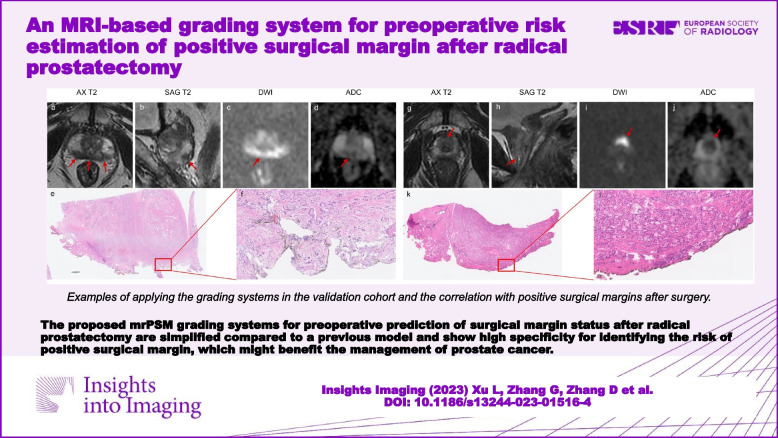

**Supplementary Information:**

The online version contains supplementary material available at 10.1186/s13244-023-01516-4.

## Introduction

Prostate cancer is the most common malignancy and the second leading cause of cancer-related death among men [1]. Radical prostatectomy (RP) is the mainstay of treatment for patients with localized prostate cancer, with the goal of cancer eradication along with preserving pelvic organ function as much as possible [2]. Positive surgical margin (PSM) in pathology after RP indicates unfavorable prognosis; specifically, PSM has been reported to be associated with an increased risk of biochemical recurrence and cancer-specific mortality [3–5], and it usually requires post-surgery therapy [6]. Preoperative prediction of PSM after RP would benefit the management of prostate cancer. For patients with a low risk of PSM, nerve-sparing surgery can be performed to preserve the functional outcomes. In patients with a high risk of PSM, recently proposed neoadjuvant treatments, especially hormonal therapy, have been demonstrated to be useful in increasing the R0 resection rates and favorably affecting the biochemical recurrence rates [7, 8].

Some preoperative clinicopathological factors, such as clinical T stage, Gleason score, percentage of positive biopsy cores, and perineural invasion detected in prostate biopsy, have been reported to correlate with the risk of PSM [3, 9–11]. In addition, pelvic anatomy is associated with PSM [12–14]. Some algorithms or nomograms have also been proposed to evaluate the risk of PSM using clinical indicators, but they lack independent validation [15].

Multiparametric magnetic resonance imaging (mpMRI) is an important imaging method for prostate cancer diagnosis and staging, and it has been reported to be useful in the prediction of extraprostatic extension (EPE) [16, 17]. Many tumor-related imaging features, such as tumor location determined by MRI, Prostate Imaging-Reporting and Data System (PI-RADS) category, length of capsular tumor contact, and distance of the lesion to the membranous urethra, have been reported to correlate with PSM [18–20]. Despite these independent imaging features, some studies have also proposed MRI-based grading systems or nomograms for preoperative prediction of PSM [10, 21]. However, these methods are complex to some extent to be used in clinical practice.

In this study, we aimed to construct a simplified scoring system using tumor-related MRI features to evaluate the risk of PSM after RP, validate the grading system in an independent cohort, and compare the model with a previously reported one.

## Materials and methods

### Patients

**Fig. 1:**
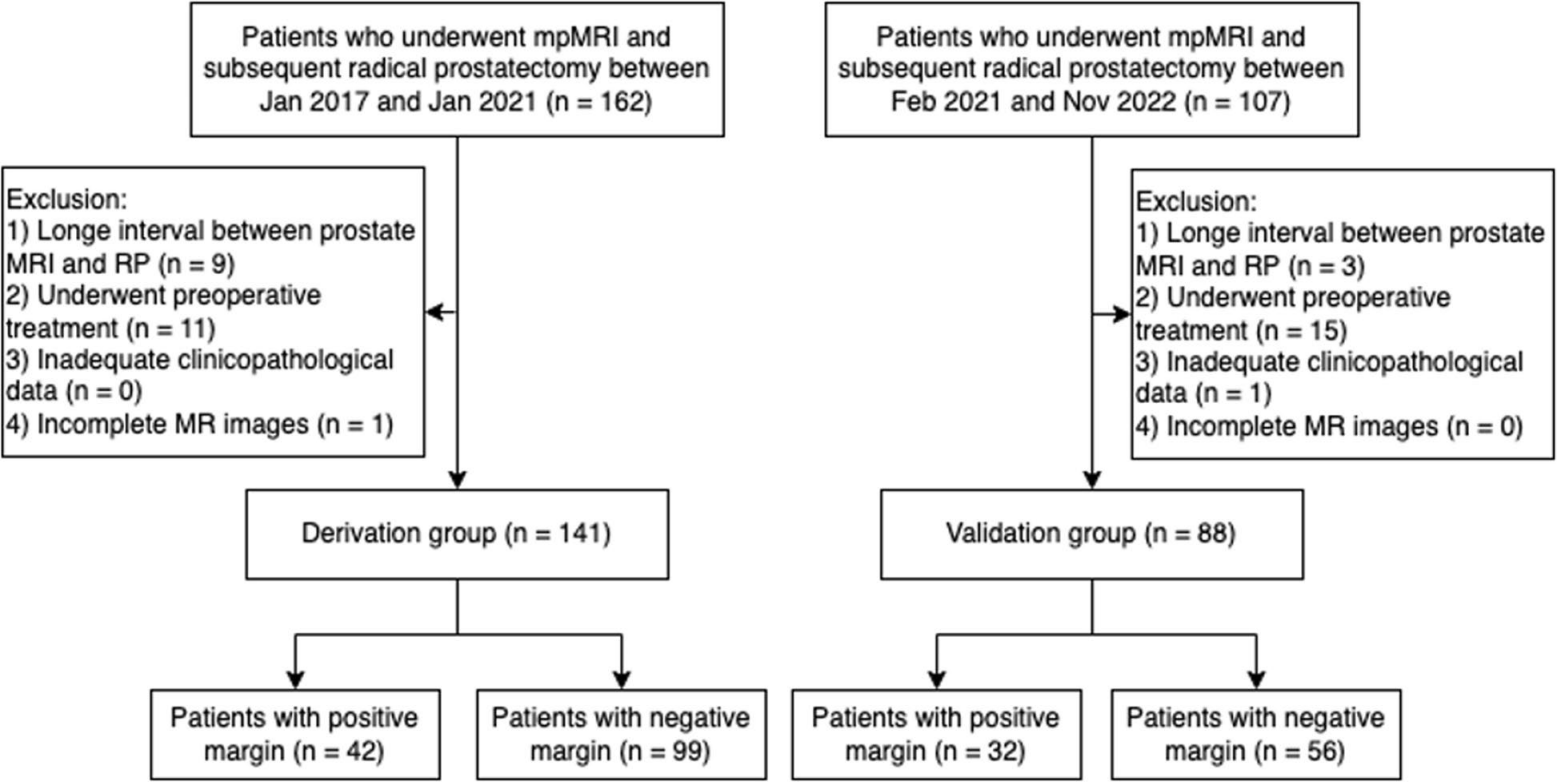
Flow diagram of patient selection for this study. mpMRI, multiparametric MRI; RP, radical prostatectomy

This retrospective study complied with HIPAA and was approved by the institutional review board of Peking Union Medical College Hospital, which waived the need for written informed consent (IRB number I-22PJ1031). Consecutive patients who had undergone preoperative prostate mpMRI followed by RP at our institution between January 2017 and January 2021 were retrospectively enrolled as the derivation group, and those between February 2021 and November 2022 were enrolled as the validation group. The exclusion criteria were as follows: (1) the interval between prostate MRI and RP was more than 6 months; (2) patients had received preoperative treatment, such as androgen-deprivation therapy, radiation therapy, or prostate-ablation therapy; (3) inadequate clinicopathological information; and (4) significant artifacts in MRI images or incomplete exams. Figure 1 shows the flow diagram of patient recruitment in this study.

The clinicopathological variables, including age, prostate-specific antigen (PSA) level, prostate volume, PSA density (PSAD), and PI-RADS score of the index lesion, were obtained from medical records.

### Image acquisition

Two 3.0T MRI scanners (GE750 [GE Healthcare] and Ingenia Elition [Philips]) were used to perform prostate mpMRI. The MRI protocol encompassed axial T1-weighted images; axial, sagittal, and coronal T2-weighted images (T2WI) (3-mm slice thickness); axial diffusion-weighted images (DWI) (3-mm slice thickness; *b*-values: 0, 100, 800, 1000, 1500, and 2000 s/mm^2^) and corresponding apparent diffusion coefficient (ADC) maps; and dynamic contrast-enhanced images (DCE) (3-mm slice thickness). The detailed MRI acquisition parameters applied in this study are shown in Supplementary S-Table 1.

### Image interpretation

The MRI scans were reviewed by one radiologist (> 1000 prostate MRI images interpreted). The following tumor-related MRI features potentially associated with PSM status were analyzed in this study: the capsule contact length (CCL) of lesions, capsular irregularity or bulge, neurovascular bundle asymmetry, obliteration of rectoprostatic angle, frank EPE visible at MRI or invasion of adjacent anatomic structures [16], and apex abutting. CCL was recorded as a continuous variable, which was defined as the maximum curvilinear length of the tumor in contact with the prostatic capsule [17]. Other imaging features were documented as either “positive” or “negative,” based on the presence or absence of the features, respectively. The definition of apex abutting was modified from the definition reported in Costa et al.’s study [22]: lesions located in the apex and abutting the most aspect of the prostate (> two quarters of the apex are involved and/or encircling the distal-most prostatic urethra). Tumors within 3 mm to the proximal membranous urethra were regarded as lesions located in the apex (these included lesions that were usually visible on the last one or two slices of the prostate apex on axial T2WI). The radiologist was blinded to postoperative surgical margin status.

After the MRI-based grading system was proposed, the previous radiologist and another radiologist (> 500 prostate MRI images interpreted) independently reviewed the images and evaluated the risk of PSM according to the grading system. All of the measurements were performed on axial T2WI in combination with coronary and sagittal T2WI with reference to high-b value DWI images and corresponding ADC map, and DCE.

### Reference standard

The RP specimens underwent routine pathological examination in accordance with the recommendations of the International Society of Urological Pathology (ISUP). The prostate surface was inked and then fixed with formalin. The entire gland was processed with a whole-mount slice thickness of 4 mm. A senior pathologist (with more than 10 years of experience in prostate pathology) evaluated the cases and recorded the ISUP grade of the lesions and the presence or absence of PSM at pathology. PSM was defined as the presence of cancer at the surgical margin [23].

### Statistical analysis

The differences in clinicopathological variables between the derivation and validation groups were assessed using the Mann–Whitney *U* test or chi-squared test as appropriate. In the derivation cohort, univariate analyses were performed to identify variables independently associated with PSM after RP. For continuous variables, the Youden *J* index was used to determine the optimal cutoff value [24] and convert the continuous variables to binary ones. To construct the grading system, the binary logistic regression method was used to select risk imaging features for PSM among the significant variables from the univariate analysis. The decision tree analysis was also used to select the risk imaging features. The grading system was then proposed by the arrangement and combination of selected risk imaging features. The receiver operating characteristic (ROC) curves of the different models were plotted, and the area under the ROC curve (AUC), diagnostic sensitivity, specificity, positive predictive value (PPV), and negative predictive value (NPV) were calculated to evaluate the diagnostic performance of these models. The DeLong test was used to compare the AUCs between different grading systems. The accuracies, sensitivities, and specificities of these methods were compared using the McNemar test. External validity was estimated from the independent validation cohort. The interreader agreement of the scoring systems was evaluated using the kappa statistic. Kappa values were interpreted as follows: 0.41–0.60, moderate agreement; 0.61–0.80, good agreement; ≥ 0.81, excellent agreement. *p* values < 0.05 were considered to be significant. All of the analyses were performed in R software (version 4.2.1; www.r-project.org).

## Results

### Patients’ demographic characteristics

A total of 141 and 88 patients were included in the derivation group (median age, 65.0 years; interquartile range [IQR], 62.0–69.0 years) and the validation group (median age, 67.0 years; IQR, 64.0–70.0), respectively. The median PSA levels were 9.00 [6.30–14.12] ng/mL in the derivation group and 8.76 [6.31–13.69] ng/mL in the validation group, and the median prostate volume was 34.0 [26.0–49.0] and 39.0 [30.0–46.0] mL in the derivation and validation groups, respectively. PSM was identified in 29.8% (42/141) of patients in the derivation group and 36.4% (32/88) of patients in the validation group. The clinicopathological characteristics between patients in the derivation group and the validation group showed no statistically significant differences (all *p* > 0.05) (Table 1).

**Table 1:** Patients’ demographic characteristics

**Variable**	**Derivation group (*n* = 141)**	**Validation group (*n* = 88)**	***p* values**
Age (year)^a^	65.0 (62.0–69.0)	67.0 (64.0–70.0)	0.097
PSA (ng/mL)^a^	9.00 (6.30–14.12)	8.76 (6.31–13.69)	0.735
Prostate volume (mL)^a^	34.0 (26.0–49.0)	39.0 (30.0–46.0)	0.124
PSAD^a^	0.28 (0.16–0.45)	0.23 (0.17–0.38)	0.124
PI-RADS (%)			0.505
2	1.4 (2/141)	0.0 (0/88)	
3	10.6 (15/141)	10.2 (9/88)	
4	44.7 (63/141)	52.3 (46/88)	
5	43.3 (61/141)	37.5 (33/88)	
Clinical T stage (%)			0.383
T1	2.8 (4/141)	0.0 (0/88)	
T2	73.8 (104/141)	76.1 (67/88)	
T3	23.4 (33/141)	23.9 (21/88)	
ISUP grade (%)			0.816
1	12.8 (18/141)	14.8 (13/88)	
2	34.0 (48/141)	29.5 (26/88)	
3	29.1 (41/141)	26.1 (23/88)	
4	11.3 (16/141)	15.8 (14/88)	
5	12.8 (18/141)	13.6 (12/88)	
Positive surgical margin (%)	29.8 (42/141)	36.4 (32/88)	0.374

Unless otherwise indicated, data are percentages

*PSAD* prostate-specific antigen density, *ISUP* International Society of Urological Pathology

^a^Data are median (interquartile range [IQR])

### Imaging feature analysis and grading system construction

In the univariate analysis, capsular irregularity or bulge, frank EPE, CCL, and apex abutting were significantly different between patients with positive and negative surgical margins (*p* = 0.002, < 0.001, < 0.001, and 0.004, respectively) (Table 2). No statistical significance was noted for neurovascular bundle asymmetry and obliteration of rectoprostatic angle (all *p* > 0.05).

**Table 2:** Univariate analysis of MRI features in the derivation group (%)

**Variable**	**PSM**	**NSM**	***p* values**
CCL (mm)^a^	20.5 (13.0–32.0)	12.0 (6.0–17.0)	< 0.001
Frank EPE	45.2 (19/42)	14.1 (14/99)	< 0.001
Capsular irregularity or bulge	71.4 (30/42)	41.4 (41/99)	0.002
Apex abutting	42.9 (18/42)	18.2 (18/99)	0.004
Neurovascular bundle asymmetry	19.0 (8/42)	9.1 (9/99)	0.168
Obliteration of rectoprostatic angle	2.4 (1/42)	2.0 (2/99)	1.000

Unless otherwise indicated, data are percentages

*CCL* capsule contact length, *EPE* extraprostatic extension, *PSM* positive surgical margin, *NSM* negative surgical margin

^a^Data are median (interquartile range [IQR])

**Fig. 2:**
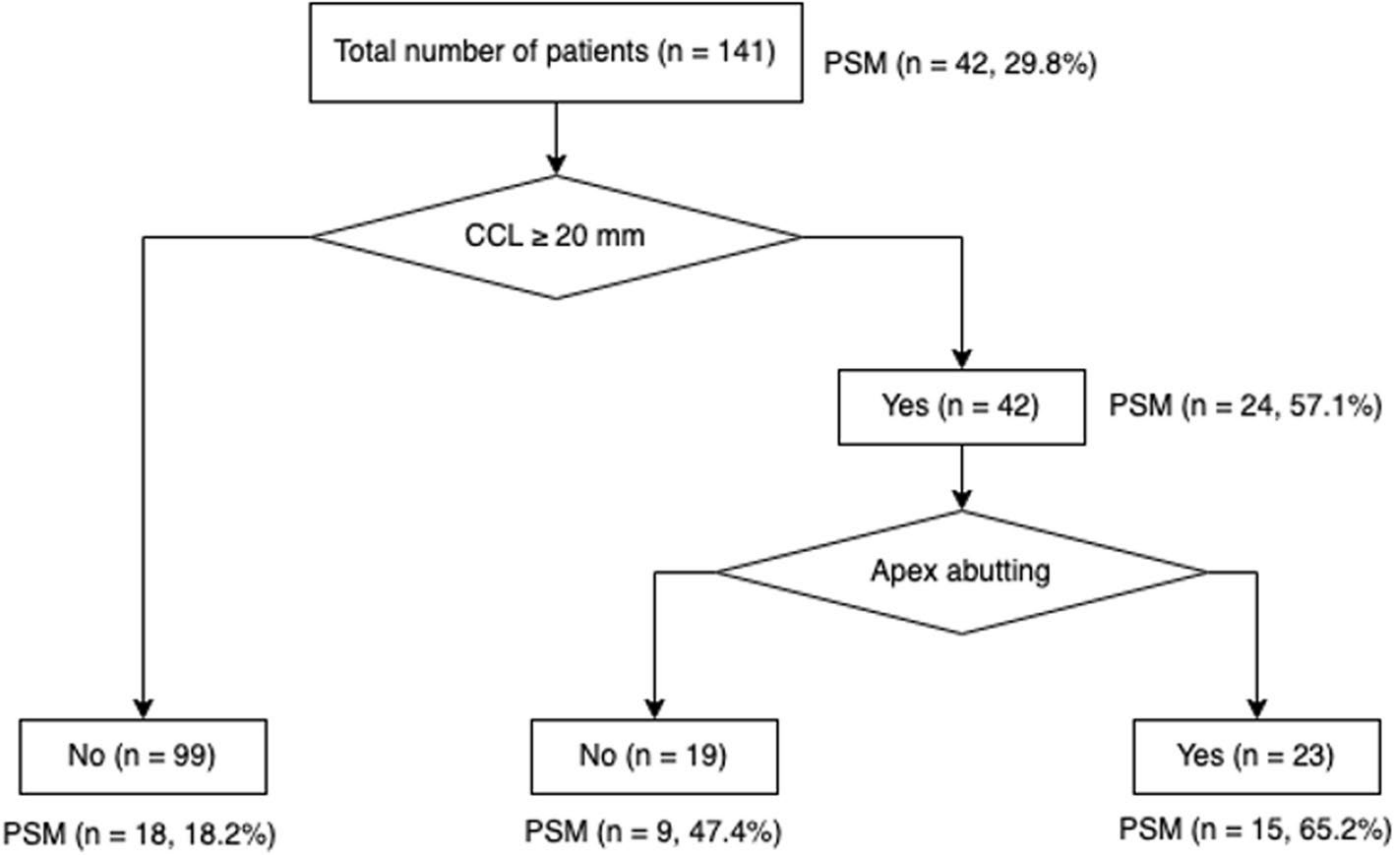
The decision tree analysis of risk MRI features. The first node indicates the capsule contact length (CCL), followed by the presence of apex abutting. PSM, positive surgical margin

The optimal cutoff value for CCL was 20 mm. Binary logistic regression showed that the CCL ≥ 20 mm was the independent risk factor for PSM (*p* = 0.039). The *β* value was 1.311 for CCL ≥ 20 mm, 0.470 for apex abutting, 0.242 for frank EPE, and 0.210 for capsular irregularity (Table 3). By decision tree analysis, the CCL was the best predictor variable for PSM. The second-best predictor variable of PSM was apex abutting (Fig. 2). In combination with the results of the two methods, the CCL and apex abutting were selected as the risk imaging features to propose the first grading system—mrPSM1:

- Grade 1, low risk of PSM: CCL < 20 mm without apex abutting;
- Grade 2, intermediate risk of PSM: CCL ≥ 20 mm or apex abutting;
- Grade 3, high risk of PSM: CCL ≥ 20 mm and apex abutting.

**Table 3:** Logistic regression of the significant imaging features in univariate analysis

**Variable**	**β**	**Wald**	**Exp (β) (95% CI)**	***p* values**
CCL ≥ 20 mm	1.311	4.249	3.710 (1.085–13.607)	0.039
Apex abutting	0.470	0.883	1.600 (0.583–4.219)	0.348
Frank EPE	0.242	0.134	1.274 (0.338–4.642)	0.715
Capsular irregularity or bulge	0.210	0.149	1.233 (0.408–3.51)	0.699

*CCL* capsule contact length, *EPE* extraprostatic extension, *CI* confidence interval

The second grading system—mrPSM2—in combination with the radiologist’s perspective was also proposed, in which frank EPE was included in grade 3, as follows:

- Grade 1, low risk of PSM: CCL < 20 mm without apex abutting;
- Grade 2, intermediate risk of PSM: CCL ≥ 20 mm or apex abutting;
- Grade 3, high risk of PSM: CCL ≥ 20 mm and apex abutting, or frank EPE.

In the derivation group, the AUC was 0.705 (0.614–0.795) for mrPSM1 and 0.713 (0.624–0.802) for mrPSM2. The accuracy, sensitivity, and specificity for mrPSM1 were 0.695, 0.643, and 0.717, respectively, and were 0.695, 0.667, and 0.707 for mrPSM2, respectively.

### Validation and comparison of mrPSM grading systems

The PSM detection rate for the different models increased with the risk category. In the validation cohort, the PSM detection rates for low-, intermediate-, and high-risk groups of mrPSM1 were 25.0%, 47.6%, and 72.7%, respectively; the values were 24.1%, 40.0%, and 68.4% for mrPSM2 and 17.9%, 42.6%, and 53.8% for Park et al.’s model. In both mrPSM1 and mrPSM2, more than half of the patients were classified as the low-risk group (63.6% [56/88] for mrPSM1 and 61.4% [54/88] for mrPSM2), while the percentage was low for Park et al.’s model (31.8% [28/88]).

**Fig. 3:**
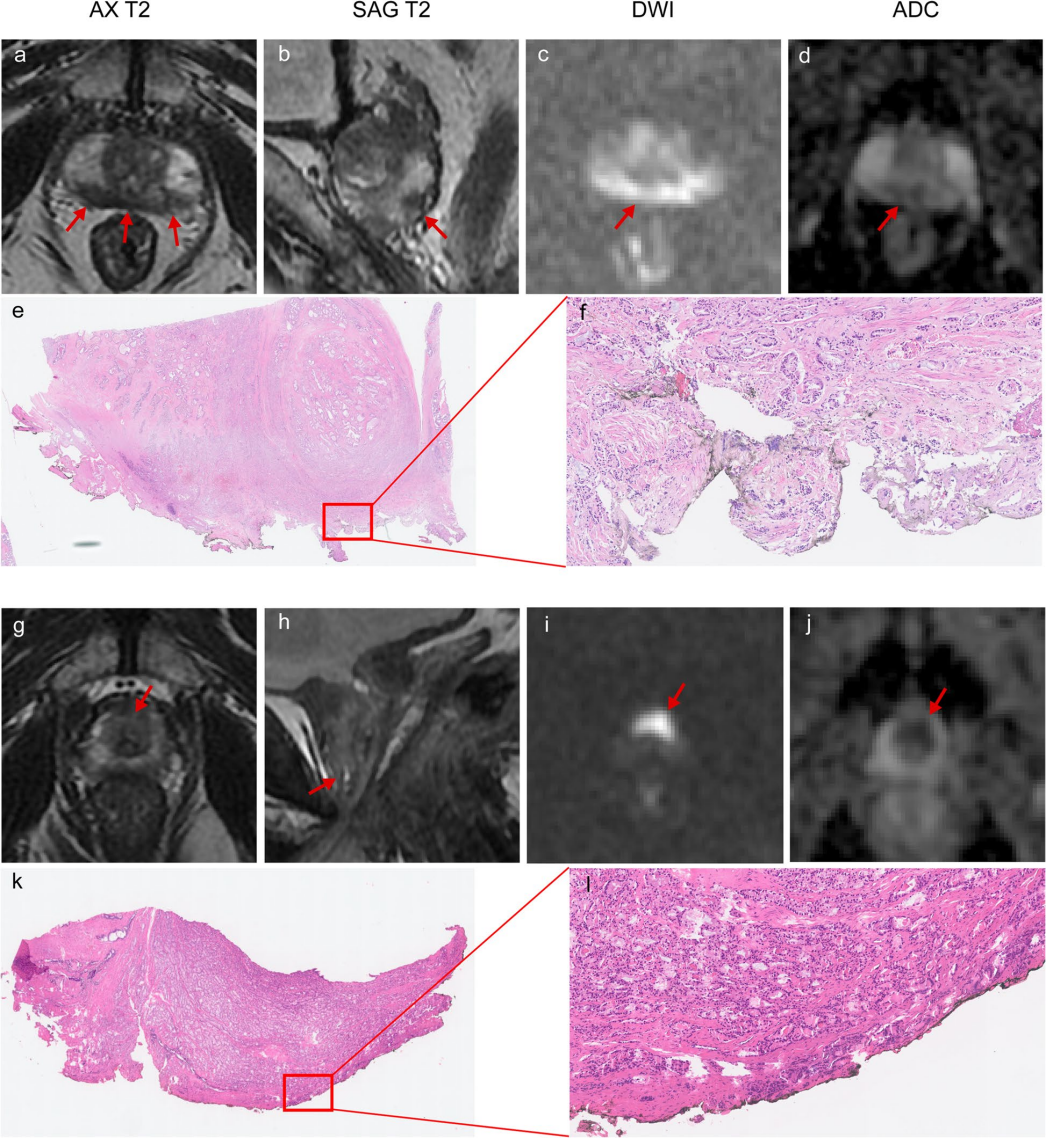
Examples of applying the grading systems in the validation cohort and the correlation with positive surgical margins after surgery. Magnetic resonance images (from the left to the right) are axial T2-weighted images (AX T2), sagittal T2-weighted images (SAG T2), diffusion-weighted images (DWI) (*b* value = 2000 s/mm^2^), and apparent diffusion coefficient (ADC) maps. **a**–**f** A 75-year-old man with prostate cancer. Images show the curvilinear contact length of the lesion in the posterior peripheral zone ≥ 20 mm (arrows). The patient was classified as grade 2 by both mrPSM1 and mrPSM2 and was considered to have intermediate risk of PSM. Whole mount pathology (**e**) and a microscopic view (**f**) of prostate shows a positive surgical margin at the right posterior. **g**–**l** A 62-year-old man with prostate cancer. MRI images show the apex lesion encircling the distal prostatic urethra (arrows) and indicating apex abutting (**g**–**j**). The patient was classified as grade 2 by both mrPSM1 and mrPSM2. Whole mount pathology (**k**) and a microscopic view (**l**) of prostate show a positive surgical margin at the apex

**Fig. 4:**
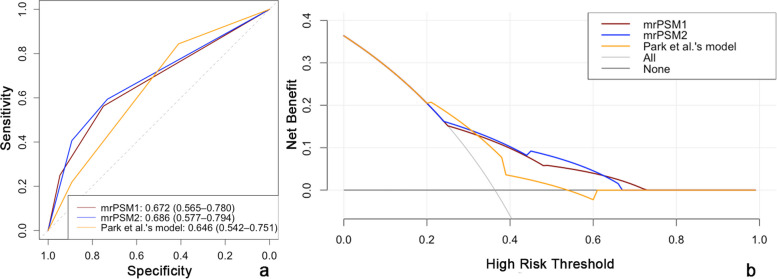
The receiver operator characteristic curves (**a**) and decision curves (**b**) of the mrPSM1, mrPSM2, and Park et al.’s model for evaluating positive surgical margins after radical prostatectomy. mrPSM1, the first MRI-based grading system for positive surgical margin; mrPSM1, the second MRI-based grading system for positive surgical margin

In the validation group, the AUC was 0.672 (0.565–0.780) for mrPSM1 and 0.686 (0.577–0.794) for mrPSM2. Both systems showed slighter higher AUC values than Park et al.’s model (AUC: 0.646 [95% CI: 0.542–0.751]), but without statistical significance (*p* = 0.566 and 0.395, respectively). The accuracy, sensitivity, and specificity for mrPSM1 and mrPSM2 were comparable (all *p* > 0.05). When compared with Park et al.’s model, our grading systems possessed significantly higher specificity (0.732–0.750 vs. 0.411, all *p* < 0.001) but lower sensitivity (0.562–0.594 vs. 0.844, all *p* < 0.05). The results are presented in Table 4. Figure 3 shows examples of how our grading systems correlate with pathological outcomes. Decision curve analysis showed that the net benefit was slightly higher for mrPSM2 (Fig. 4).

**Table 4:** Performance of mrPSM grading systems in the derivation and validation groups

	**Derivation group**		**Validation group**			***p* values**	
**Grading systems**	**mrPSM1**	**mrPSM2**	**mrPSM1**	**mrPSM2**	**Park et al.’s system**	**mrPSM1 vs. Park et al**	**mrPSM2 vs. Park et al**
Low risk (%)^a,b^	17.4 (15/86)	16.7 (14/84)	25.0 (14/56)	24.1 (13/54)	17.9 (5/28)	-	-
Intermediate risk (%)^a,b^	37.5 (12/32)	31.8 (7/22)	47.6 (10/21)	40.0 (6/15)	42.6 (20/47)	-	-
High risk (%)^a,b^	65.2 (15/23)	60.0 (21/35)	72.7 (8/11)	68.4 (13/19)	53.8 (7/13)	-	-
AUC	0.705 (0.614–0.795)	0.713 (0.624–0.802)	0.672 (0.565–0.780)	0.686 (0.577–0.794)	0.646 (0.542–0.751)	0.566	0.395
Cut-off value	≥ 2	≥ 2	≥ 2	≥ 2	≥ 2	-	-
Accuracy	0.695 (0.692–0.698)	0.695 (0.692–0.698)	0.682 (0.677–0.687)	0.682 (0.677–0.687)	0.568 (0.563–0.574)	0.089	0.078
Sensitivity	0.643 (0.498–0.788)	0.667 (0.524–0.809)	0.562 (0.391–0.734)	0.594 (0.424–0.764)	0.844 (0.718–0.970)	0.008	0.013
Specificity	0.717 (0.628–0.806)	0.707 (0.617–0.797)	0.750 (0.637–0.863)	0.732 (0.616–0.848)	0.411 (0.282–0.540)	< 0.001	< 0.001
PPV	0.491 (0.359–0.623)	0.491 (0.361–0.621)	0.562 (0.391–0.734)	0.559 (0.392–0.726)	0.450 (0.324–0.576)	-	-
NPV	0.826 (0.745–0.906)	0.833 (0.754–0.913)	0.750 (0.637–0.863)	0.759 (0.645–0.873)	0.821 (0.680–0.963)	-	-

*AUC* area under the receiver operating characteristic curve, *PPV* positive predictive value, *NPV* negative predictive value, *mrPSM1* the first MRI-based grading system for positive surgical margin, *mrPSM1* the second MRI-based grading system for positive surgical margin

^a^Data are the percentage of PSM

^b^For mrPSM1 and mrPSM2, patients who were classified into grade 1, grade 2, and grade 3 were considered to have low, intermediate, and high risk of PSM, respectively; for Park et al.’s system, patients with risk score of 0–2, 3–5, and 6–7 were considered to have low, intermediate, and high risk of PSM, respectively

Regarding the interreader agreement of the overall assessment category, the kappa value was 0.764 for mrPSM1 and 0.776 for mrPSM2, with both indicating good agreement. When category ≥ 2 was considered positive, the agreement was also good for both versions (kappa value: 0.810 for mrPSM1 and 0.780 for mrPSM2).

## Discussion

Our study constructed MRI-based grading systems for preoperative prediction of PSM in patients who underwent RP and validated them in an independent cohort. By combining logistic regression and decision tree analysis, we proposed the first MRI-based grading system—mrPSM1—and a further modification of the grading system performed by integrating the radiologist’s knowledge yielded the second MRI-based grading system—mrPSM2. Both grading systems showed the feasibility of predicting PSM with AUCs of 0.705 and 0.713 in the derivation group and 0.672 and 0.686 in the validation group. The proposed MRI-based grading systems showed significantly higher specificity than Park et al.’s system. The mrPSM2 seemed to outperform mrPSM1 and Park et al.’s system with higher net benefit.

A positive margin after RP could be related to the unresectable extracapsular extension tumor or surgical reasons. Our proposed grading system contained both imaging features related to anatomical information (apex abutting) and pathological EPE (CCL and frank EPE) and, therefore, possess the validity to predict the risk of PSM. A PSM occurs most commonly at the prostatic apex because of anatomical and surgical reasons [19]. Preoperative MRI-identified tumor at the apex is a significant factor for PSM [25–27]. Costa et al. [22] flagged prostate lesions abutting the apical-most gland and/or encircling the distal-most prostatic urethra in the structured report on MRI and found a significantly higher proportion of apical PSMs in the flagged group. Quentin et al. [18] highlighted the importance of assessing the risk of PSM at the apical urethra or at the capsule separately. Their study showed that the capsule (57%, 40/70) and the apical urethra (22%, 15/70) were the two most common sites of PSM. In addition, for PSM at the apical urethra, distance to the membranous urethra (UD) ≤ 3.5 mm reached an accuracy of 95%. Considering the varied definition of apex involvement between studies, we combined the distance information and the lesion feature to generate the apex abutting feature. This feature was demonstrated to be useful in the prediction of PSM. Additionally, this qualitative feature seems to be easier to be recorded than quantitative measurement. Our research supported previous studies that have demonstrated that CCL is an important risk factor for PSM [18, 21]. For a more convenient interpretation, we converted the continuous variable to a binary one. The cutoff value for CCL in our study varied from what Quentin et al. reported [18] but not by much. Frank EPE observed on MRI has widely been investigated and demonstrated as an independent risk factor for PSM [20, 27, 28], because this feature is demonstrated to be correlated with pathological EPE which could be difficult to be completely resected. Therefore, this risk feature was integrated into the mrPSM2 in this study. Although no statistical difference was noted in the AUC, sensitivity, specificity, and accuracy between the mrPSM1 and mrPSM2, the mrPSM2 was preferred for clinical practice with higher benefits shown by the decision curve analysis. PI-RADS score is another imaging feature that has widely been investigated for predicting PSM [20, 21]. According to the PI-RADS guideline [29], lesions with diameters ≥ 1.5 cm or definite EPE should be upgraded from PI-RADS score 4 to score 5, and for lesions at the capsule, a larger tumor size might be correlated with longer CCL and increased the risk of EPE. Although the PI-RADS score might contain information on tumor-capsule correlation, the PI-RADS score itself could not reflect the contact of the lesion with the prostate capsule directly [30] or indicate the tumor location; therefore, we chose other tumor-related imaging features for analysis rather than the PI-RADS score.

Converting the independent risk features to a three-category grading system would facilitate the structured risk evaluation of PSM as well as the education and popularization of it in the future. Our MRI-based grading system is simplified with only three imaging features that need to be recorded. By calculating the interreader agreement between the experienced (> 1000 prostate MRI images interpreted) and less experienced radiologist (> 500 prostate MRI images interpreted) for the mrPSM2, the kappa value was 0.776 for the overall assessment category and 0.780 for the predefined positive category (grade ≥ 2), which indicates a good agreement. In addition, because our grading systems rely on MRI, the quality of MRI images should not be ignored. Presently, Prostate Imaging Quality (PI-QUAL) has been proposed for the quality control of prostate MRI [31]. Studies have shown that assessing image quality using PI-QUAL is helpful in the evaluation of EPE, with improved accuracy in high-image-quality studies [32]. Although no examination was excluded due to inadequate image quality in our study, further analyzing the influence of PI-QUAL on the evaluation of PSM risk is needed.

Some previous studies have also tried to propose MRI-based grading systems for PSM. Chen et al. [10] developed an MRI-based tool for PSM, with AUC, sensitivity, and specificity of 0.735, 57%, and 88%, respectively. However, their model was based on complex quantitative imaging features from DWI, intravoxel incoherent motion model, and diffusion kurtosis imaging, which hampered its application in a wider patient population. Park et al. [21] proposed their scoring system based on the following variables: tumor-capsule contact length, PI-RADS category, and tumor located at the apex and/or posterolateral side. In our validation cohort, the AUC of their model (0.646) was slightly lower than that of our grading systems (0.672–0.686) without statistical significance. Park et al.’s model heavily weights the PI-RADS score, resulting in a significant portion of patients with intermediate- to high-risk PSM. It is worth noting that in both Park et al.’s and our validation cohort, the use of Park et al.’s system classified the majority of patients into the intermediate-risk group (56.0% and 53.4%, respectively), which is an equivocal classification and might put urologists in a dilemma as to how to deal with these cases. By using the mrPSM2 grading system, most patients were classified with low risk of PSM (61.4% [54/88]) in the validation group and could maintain the surgery, while patients with high risk of PSM might be recommended neoadjuvant therapy with a PSM detection rate of 68.4% (13/19). Although our grading systems possessed risk stratification possibility for PSM with high specificity, the sensitivity of the grading systems is relatively low because the majority of patients were classified into the low-risk group, which means a portion of patients with PSM could not be identified by our systems. Therefore, further clinicopathological characteristics might need to be taken into consideration for patients who were classified with low risk of PSM by our grading system.

There are some limitations to this study. First, the retrospective nature of this study may have introduced some selection bias. Further validation of this grading system in prospective cohorts is necessary. Second, the grading system is merely based on MRI features. For a more precise evaluation of PSM, integrating our grading system with clinical risk factors and surgeon’s influence is recommended in future studies [33]. In addition, artificial intelligence and radiomics also showed the potential of predicting PSM and might be further acknowledged [34, 35]. Finally, considering the multifocal feature of PSM, per-lesion analysis needs to be carried out in upcoming studies.

In conclusion, the proposed mrPSM grading systems for postoperative surgical margin status prediction are simplified and possess the feasibility in risk stratification; they show high specificity for identifying patients with the risk of PSM and potentially providing benefits for the management of patients with prostate cancer.

### Supplementary Information

**Additional file 1: S-Table 1.** Sequence parameters for prostate multiparametric MRI.

## Data Availability

The datasets used and/or analyzed during the current study are available from the corresponding author on reasonable request.

## Abbreviations

AUC: Area under the curve
CCL: Capsule contact length
EPE: Extraprostatic extension
ISUP: International Society of Urological Pathology
mpMRI: Multiparametric magnetic resonance imaging
NPV: Negative predictive value
PI-RADS: Prostate Imaging-Reporting and Data System
PPV: Positive predictive value
PSA: Prostate-specific antigen
PSAD: Prostate-specific antigen density
PSM: Positive surgical margin
ROC: Receiver operating characteristic
RP: Radical prostatectomy
UD: Distance to the membranous urethra
