# Supplementary material for: An MRI-based grading system for preoperative risk estimation of positive surgical margin after radical prostatectomy
Source: Insights Imaging. 2023 Oct 23;14:178. doi: 10.1186/s13244-023-01516-4 (PMC10593712; doi:10.1186/s13244-023-01516-4)
Supplement: Supplementary file 1 — Additional file 1: S-Table 1. Sequence parameters for prostate multiparametric MRI. [file 13244_2023_1516_MOESM1_ESM.docx]

**An MRI-based grading system for preoperative risk estimation of positive surgical margin after radical prostatectomy**

**ELECTRONIC SUPPLEMENTARY MATERIAL**

**S-Table 1** Sequence parameters for prostate multiparametric MRI

| **Parameters** | **T2WI** | **DWI** | **DCE-MRI** |
| --- | --- | --- | --- |
| Sequence | FRFSE, TSE | SE-EPI, SE | 3D-GRE |
| TR/TE (ms) | 4400/108, 4000/100 | 2800/69, 4000/65 | 3.0/1.3, 3.5/1.0 |
| Echo train length | 19 | 1, 47 | NA |
| Field of view (cm × cm) | 26 × 26, 20 ×20 | 36 × 36, 16 × 16 | 40 × 40, 35 × 35 |
| Matrix size | 288 × 192, 236 × 184 | 128 × 96, 64 × 64 | 320 × 192, 232 × 219 |
| Thickness (mm) ^*^ | 3.0 | 3.0 | 3.0 |
| Other |  | b values = 0, 100, 800, 1000, 1500, 2000 s/mm^2^ | Temporal resolution < 10s, and total scan time of 5 min |

Note—TR = repetition time, TE = time echo, FRFSE = fast relaxation fast spin echo, SE-EPI = spin-echo echo-planar imaging, 3D-GRE = 3D-gradient echo, DCE = dynamic contrast-enhanced MRI.

^*^ Without gap.
